# Gluteal Compartment Syndrome After Prolonged Immobilization in Drug Abusers

**DOI:** 10.7759/cureus.9847

**Published:** 2020-08-18

**Authors:** Anupam K Gupta, Monica I Burgos, Miguel Lopez-Viego, Nir Hus

**Affiliations:** 1 Minimally Invasive Surgery, University of Miami Hospital, Miami, USA; 2 Internal Medicine, Universidad Autonoma de Guadalajara, Guadalajara, MEX; 3 Surgery, Bethesda Memorial Hospital, Boynton Beach, USA; 4 Surgery, Delray Medical Center, Delray Beach, USA; 5 Surgery, Florida Atlantic University, Boca Raton, USA

**Keywords:** opiods, gluteal compartment syndrome, drug abuse, atypical presentation

## Abstract

Background

There has been an increasing incidence of drug abuse patients presenting with rhabdomyolysis after prolonged immobilization. Our study was to assess etiology and management challenges with patients presenting with gluteal compartment syndrome after drug abuse.

Methodology

We did a retrospective analysis of five patients who presented with gluteal compartment syndrome secondary to drug abuse over one year.

Results

We had a 100% association with rhabdomyolysis and acute renal injury necessitating hemodialysis. There was a frequent association with the involvement of additional compartments like thigh and leg.

Conclusion

Patients with drug overdose can present with unusual compartment syndrome involvement like the gluteal compartment. Compartment syndrome is a surgical emergency and needs multidisciplinary involvement.

## Introduction

Acute compartment syndrome manifests by an increase in intracompartmental pressure higher than that of perfusion pressure [1,2]. Compartment syndrome is an acute limb/life-threatening situation that needs emergent surgical intervention. There has been an increasing trend of drug abuse, and we have had a few patients arrive to the emergency room after passing out secondary to drug abuse presenting with compartment syndrome and rhabdomyolysis. In a fraction of these patients, we found the significant involvement of gluteal compartment syndrome, and a retrospective study performed at our center.

## Materials and methods

We introduce a retrospective case series of patients presenting with gluteal compartment syndrome secondary to a drug overdose. Over one year from January 2019 to January 2020, we had multiple patients present with compartment syndrome of the limbs secondary to drug overdose and prolonged immobilization. The patients of this study are from Bethesda Hospital East in Boynton Beach, Florida.

Electronic medical records from the hospital were used to gather data. We evaluated the patient's location of immobilization and duration of immobilization based on history. Motor involvement implied patient had restriction of hip movement. Sensory involvement implied if the patient could not do two-point discrimination or complained of paresthesia. The patient's diagnosis of compartment syndrome was based on clinical examination/imaging in the form of computed tomography, intraoperative findings, and evidence of rhabdomyolysis with elevated creatine kinase (CK).

Inclusion criteria included patients who were immobile after drug overdose presenting with compartment syndrome of the buttock and possibly other compartments. 

Exclusion criteria included patients who had only limb compartment syndrome, excluding the buttocks, and compartment syndrome secondary to other etiology.

Analysis

In terms of outcomes of the duration of immobilization, compartments involved, renal failure, and need for hemodialysis, it was measured in terms of absolute numbers and percentages.

## Results

Data from patients who presented with gluteal compartment syndrome at Bethesda Hospital in Boynton Beach were analyzed (Table 1). Over one year, we had a total of five patients who had involvement of the gluteal compartment. The age range was 29-43 years, with a mean age of 35.4 years. All five of our patients were found immobile on hard surfaces for a duration between 8 and 14 hours with a mean duration being 10.4 hours. All of the patients arrived in the emergency room with the help of emergency service; there was a 100% involvement of motor function (reduced mobility of hip joint) by the patient.

**Table 1:** Patient Data Sl no. = serial number; M = male; PMH = past medical history; CK = creatine kinase value in brackets at time of arrival; HD = need for hemodialysis; Mt/S = motor/sensory involvement, Y = yes involved; N = not involved.

Sl.No.	Age/sex	Location/duration hours	Additional compartments	PMH	Diagnosis	HD	Mt/S
1	29/M	Bathroom/8 hours	Right gluteal + right thigh and leg	None	Clinical + CK (44200)	Y	Y/Y
2	33/M	Bathroom/14 hours	Left gluteal + left thigh and leg	None	CT + CK (73400)	Y	Y/Y
3	30/M	Bathroom/10 hours	Left gluteal + left thigh and leg	None	CT + CK (45000)	Y	Y/Y
4	42/M	Stair case/12 hours	Left gluteal + left thigh and leg	None	Clinical + CK (54600)	Y	Y/Y
5	43/M	Bathroom/8 hours	Isolated left gluteal	None	Clinical + CT + CK (20947)	Y	Y/Y

**Figure 1:**
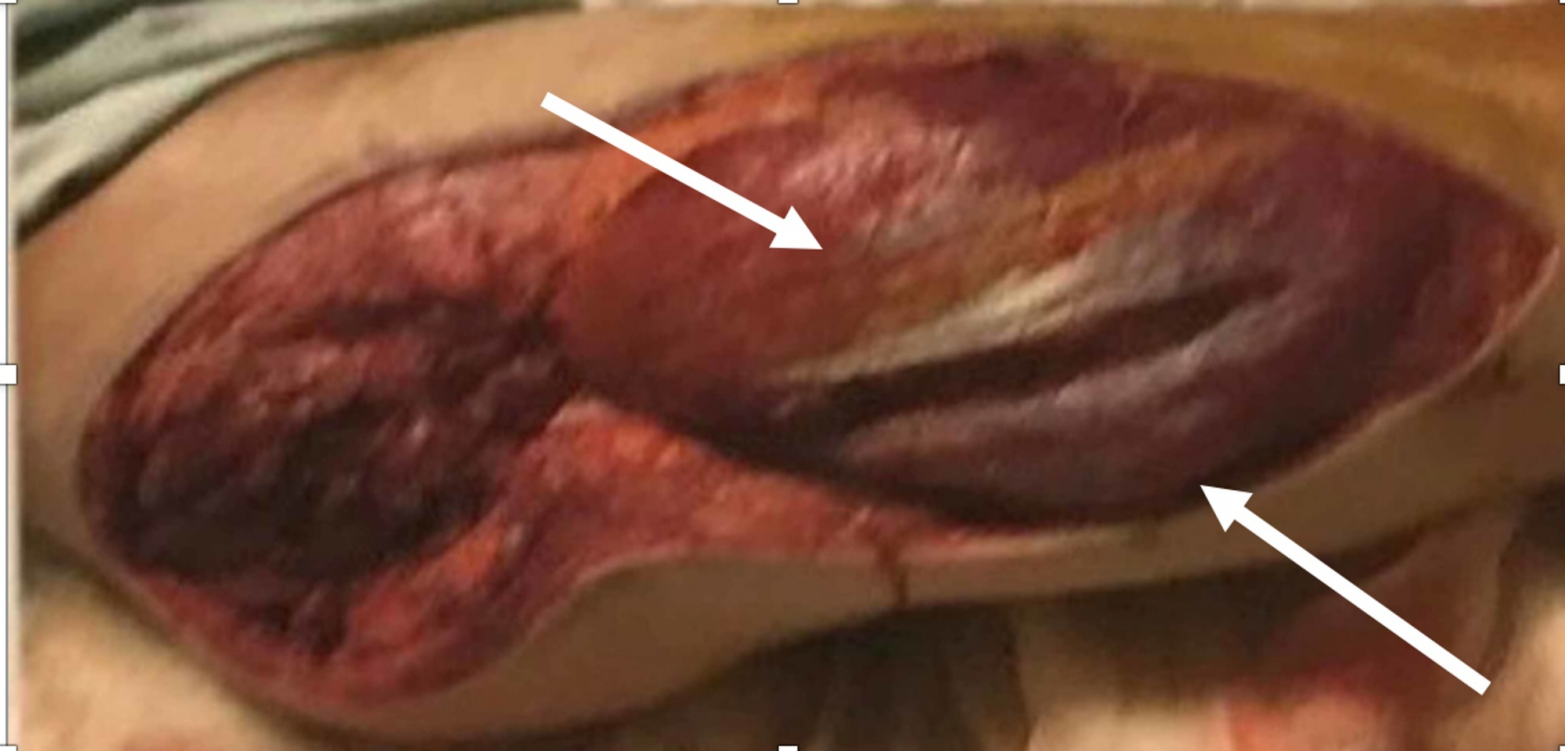
Muscle bulge after fasciotomy of gluteal compartment

Approximately 100% of the patients had sensory involvement with the inability to do two-point discrimination and paresthesia. Three out of the five patients (60%) were subjected to additional CT by the emergency room physicians before being evaluated by the surgical team. On clinical examination, all patients presented with swelling, pain, and paresthesia, which are characteristics of compartment syndrome. In addition, all of the patients had evidence of an elevated CK suggestive of rhabdomyolysis, which had a direct correlation with the duration of immobilization and extent of compartments involved. All the patients were taken emergently for a decompressive fasciotomy (Figure 1); we had a 100% correlation with acute renal injury despite forced alkaline diuresis, which the patients subsequently needed hemodialysis to correct hyperkalemia. The patients needed subsequent surgery for closure of the fasciotomy wound, which could be either done primarily with a split skin graft or a combination. Post fasciotomy with aggressive physical therapy and wound care, patients subsequently had a return of limb function and renal function. On follow-up at a few months intervals, none of the patients needed hemodialysis.

## Discussion

Compartment syndrome is a condition that can develop when there is an increase in interstitial pressure that builds up in the fascia compartment [1-3]. This sheath limits the expansion and causes an increase in the volume of its contents, leading to an increase in compartment pressures [3]. Signs and symptoms, as a result, are a pain out of proportion, pallor, paresthesia, paralysis, and edema. Pronounced neurological symptoms with motor deficits, absent pulses, and poikilothermia occur later on and usually indicate irreversible damage [4]. One of the cardinal symptoms is, but not limited to, pain out of proportion [5]. Upon clinical examination, one can feel tense, edematous compartments, and pain on passive stretch [3]. However, pain may be an unreliable symptom in some cases as it is subjective and variable. The diagnosis of compartment syndrome is due to subjective and objective findings. Other than the above mentioned subjective findings, we can measure compartment pressure. This pressure, also known as the compartment delta pressure, is the difference between the diastolic blood pressure and the intracompartmental pressure [1]. Various devices are used to detect this intracompartmental pressure, which, in essence, uses a needle to be inserted in a muscle compartment and injection of saline solution to measure the resistance in the muscle [1,6]. The standard pressure falls between 0 and 8 mmHg [1]. Pulse pressure "ΔP" (diastolic blood pressure - intramuscular pressure) with a value more than 30 mmHg is suggestive of inadequate perfusion and should correlate with the clinical situation [3,6]. Acute compartment syndrome needs an immediate fasciotomy [7]. Fasciotomy incisions should be large enough to allow adequate decompression of all involved compartments [3,7]. A delay of more than six hours in diagnosing can lead to irreversible muscle damage or death. 

As drug abuse continues to be a rising concern in the United States, we can expect an increase in patients developing uncommon compartment involvement, as seen in our patients [8-11]. Drugs of abuse, such as opioids, alcohol, and other recreational drugs, can cause prolonged immobilization in prone, lateral, or sitting positions, which can result in muscle compression and ischemia in the compartment [9]. Drug-related compartment syndrome has not been reported much. The abuse of drugs can have severe ramifications on a person's physical health. The effects of opioids and recreational drugs may include decreased central nervous system function and respiratory function, skin flushing, dry mouth, and nausea [8,10]. Other more severe effects of opioids include peripheral nervous system injury, rhabdomyolysis, muscle rigidity, myoclonus, and compartment syndrome [10].

It is crucial to create awareness of the development of unusual compartment syndromes, such as in the gluteal region, and arm region [6]. The most common recognized location for the development of the syndrome involves the thigh [2]. Both the gluteal and arm regions have three compartments in which the anatomical constraints do not accommodate excessive swelling and edema, rendering it susceptible to muscle ischemia in patients with drug dependence [6,12]. Patients, when consuming large amounts of opioids/substance of abuse potential, lose voluntary movement and consciousness [8]. The loss of consciousness is the major contributing factor that leads the patients to have no control over how they land, causing them to immobilize with unorthodox stances on hard surfaces. Prolonged immobilization with compression promotes increased intracompartmental pressure in the muscle in these unusual locations [13]. Continued physical examination becomes very important for the early recognition of these uncommon syndromes [10]. The literature review revealed few reports related to the occurrence of gluteal compartment syndrome secondary to prolonged immobilization after a drug overdose [12,13]. Compartment syndrome taking place in the upper limb is also a rare presentation, as mentioned above [14,15]. Literature has shown its occurrence after infiltration of the shoulder following injection, tourniquet use, and extravasation of fluids during pressure infusion [14]. 

A severe complication of compartment syndrome is rhabdomyolysis [15]. Compartment syndrome contributes to the development of rhabdomyolysis, muscle groups are in rigid fas­cial compartments, and swelling by the third spa­cing of fluids with a traumatized muscular tissue can result in increased intra­compartmental pressure that can cause addi­tional damage by compromising both venous and arterial blood flow [2,16,17]. Muscle damage results in the leakage of intracellular muscle toxins and proteins such as myoglobin, CK, and electrolytes such as potassium into the bloodstream [16,17]. The primary mechanism of muscular injury is through a reperfusion process and vasoconstriction [18]. Prompt recognition and early intervention are vital. Full recovery is possible with early diagnosis and treatment of the many complications that can develop in patients with this syndrome. 

Patients with rhabdomyolysis can present with muscular weak­ness, generalized or localized myalgia, edema, and dark brown urine. Myoglobinuria makes the urine appear dark colored [19]. Hyperkalemia secondary to rhabdomyolysis can cause cardiac arrhythmias and death. Treatment of rhabdomyolysis comprises the initial stabilization of the patient, along with aggressive fluid therapy. Alkalization of urine reduces the formation of myo­globin casts in renal tubules [18]. By aggressive hydration at a rate of 1.5 L/h, a urinary output goal of 200 mL/h, urine pH >6.5, and plasma pH <7.5 can prevent renal injury; however, once there is oliguric renal injury, aggressive hydration can lead to fluid overload causing increasing edema and breathlessness. Sufficient monitoring is also crucial, since complications such as hypernatremia (fluid shift to intravascular) and hyperosmolality may occur [17]. Despite aggressive and early treatment in patients with rhabdomyolysis, almost all develop acute renal injury resulting in severe acidosis and hyperkalemia requiring emergent hemodialysis [18]. Continuous venovenous hemofiltration (CVVH), if diuresis fails, can remove the excessive serum-free myoglobin and electrolytes.

Rhabdomyolysis releases CK, which is an important marker that can be used to evaluate muscle injury [15]. It is the most sensitive and specific indicator of myocyte injury. The extent of rhabdomyolysis correlates to CK and glomerular filtration rate (GFR). Under physiologic conditions, myoglobin circulates in the blood bound to plasma globulins, which are at a level of 0-0.003 mg/dL [20]. Once serum myoglobin levels reach a level above 0.5-1.5 mg/dL, serum protein binding capacity is overwhelmed, and myoglobin gets excreted in the urine. Although myoglobin usually excreted by the glomerulus and excreted in the urine, the presence of large amounts of myoglobin in renal tubules leads to precipitation and tubular obstruction [19,20].

## Conclusions

Gluteal compartment syndrome is an unusual compartment to be involved that can occur as a result of prolonged immobilization secondary to a drug overdose. It is a life-threatening situation requiring identification and urgent surgical decompressive fasciotomy of gluteal and associated compartments. Hemodialysis is emergently warranted in patients who develop acute renal injury, severe hyperkalemia, and rhabdomyolysis. There is a direct correlation to the duration of immobility, compartments involved, CK levels, and motor and sensory impairment.
